# Giant peritoneal hydatid cyst causing pelvic venous congestion

**DOI:** 10.1590/0037-8682-0349-2022

**Published:** 2022-10-24

**Authors:** Emine Izgi, Hayri Ogul, Yener Aydin

**Affiliations:** 1 Medizinisches Versorgungszentrum Meine Radiologie Tuttlingen GmbH, Department of Radiology, Tuttlingen, Germany.; 2 Duzce University, Medical Faculty, Department of Radiology, Duzce, Turkey.; 3 Ataturk University, Medical Faculty, Department of Thoracic Surgery, Erzurum, Turkey.

**FIGURE 1:**
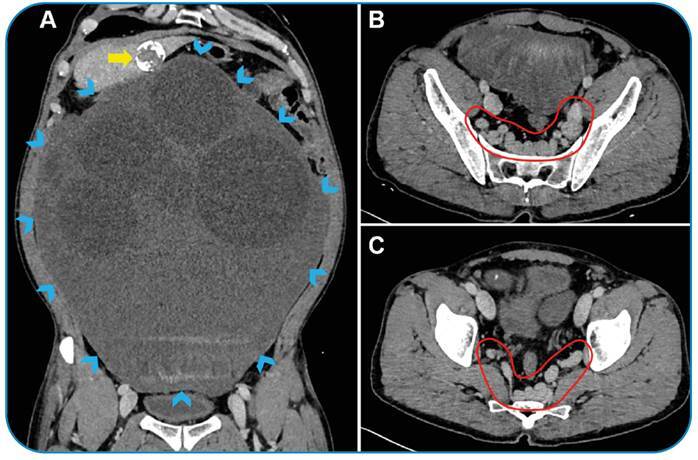
Coronal abdominal computed tomography (CT) scan **(A)** reveals a giant peritoneal hydatid cyst (arrowheads) and a calcified hepatic cyst (yellow arrow). Axial pelvic CT scans **(B and C)** reveal multiple dilated and tortuous pelvic venous structures (circle) in the presacral area.

A 53-year-old woman without a history of chronic disease was admitted to our hospital. On admission, the patient recounted a history of progressive abdominal distension and pelvic pain over the preceding 18 months. She had no history of systemic disease or abdominal trauma. On physical examination, a large, round abdominal mass was palpable. Abdominopelvic computed tomography revealed a giant peritoneal hydatid cyst and tortuous pelvic venous structures associated with compression by the peritoneal cyst (Figure 1).

A hydatid cyst is a parasitic disease caused by the larval form of *Echinococcus granulosus* ^1^. The liver is a vital organ that interacts with other organs^1^ ^,^ ^2^. Peritoneal cysts, which are of a secondary origin, occur after the rupture of the primary hepatic hydatid cyst. A study has reported that peritoneal cysts develop in approximately 5-14% of patients with liver hydatid cysts^3^. Peritoneal hydatid cysts vary in number and can reach dimensions that cause abdominal distension or obstruction^3^. Pelvic venous congestion secondary to a giant peritoneal hydatid cyst is an unusual complication.
